# Transcriptomic analyses of the HPG axis-related tissues reveals potential candidate genes and regulatory pathways associated with egg production in ducks

**DOI:** 10.1186/s12864-022-08483-y

**Published:** 2022-04-08

**Authors:** Xiping Yan, Hehe Liu, Jiwei Hu, Xingfa Han, Jingjing Qi, Qingyuan Ouyang, Bo Hu, Hua He, Liang Li, Jiwen Wang, Xianyin Zeng

**Affiliations:** 1 A Department of Engineering and Applied Biology, College of Life Science, Sichuan Agricultural University, Ya’an, Sichuan, 625014 People’s Republic of China; 2 Farm Animal Genetic Resources Exploration and Innovation Key Laboratory of Sichuan Province, Sichuan Agricultural University, Chengdu, Sichuan 611130 People’s Republic of China

**Keywords:** Ducks, Transcriptome, HPG axis, DEGs, Reproductive regulation, Egg production

## Abstract

**Background:**

Egg production is one of the most important economic traits in the poultry industry. The hypothalamic-pituitary–gonadal (HPG) axis plays an essential role in regulating reproductive activities. However, the key genes and regulatory pathways within the HPG axis dominating egg production performance remain largely unknown in ducks.

**Results:**

In this study, we compared the transcriptomic profiles of the HPG-related tissues between ducks with high egg production (HEP) and low egg production (LEP) to reveal candidate genes and regulatory pathways dominating egg production. We identified 543, 759, 670, and 181 differentially expressed genes (DEGs) in the hypothalamus, pituitary, ovary stroma, and F5 follicle membrane, respectively. Gene Ontology (GO) analysis revealed that DEGs from four HPG axis-related tissues were enriched in the "cellular component" category. Kyoto Encyclopedia of Genes and Genomes (KEGG) enrichment analysis indicated that the neuroactive ligand-receptor interaction pathway was significantly enriched based on DEGs commonly identified in all four HPG axis-related tissues. Gene expression profiles and Protein–Protein Interaction (PPI) network were performed to show the regulatory relationships of the DEGs identified. Five DEGs encoding secreted proteins in the hypothalamus and pituitary have interaction with DEGs encoding targeted proteins in the ovary stroma and F5 follicle membrane, implying that they were these DEGs might play similar roles in the regulation of egg production.

**Conclusions:**

Our results revealed that neuroactive ligand-receptor interaction pathway and five key genes(*VEGFC*, *SPARC*, *BMP2*, *THBS1*, and *ADAMTS15*) were identified as the key signaling pathways and candidate genes within the HPG axis responsible for different egg production performance between HEP and LEP. This is the first study comparing the transcriptomic profiles of all HPG axis-related tissues in HEP and LEP using RNA-seq in ducks to the best of our knowledge. These data are helpful to enrich our understanding of the classical HPG axis regulating the egg production performance and identify candidate genes that can be used for genetic selection in ducks.

**Supplementary Information:**

The online version contains supplementary material available at 10.1186/s12864-022-08483-y.

## Background

Poultry eggs are one of the best nutrition sources for human health, consisting of rich vitamins, minerals, and high-quality proteins. There is no doubt that poultry eggs occupy a vital position in the dietary composition of humans. The breeds with higher egg-laying performance have advantages in advancing the breeding programs and reducing the feed costs. Thus, selection toward high egg production has greatly contributed to the genetic improvement of commercial lines in the past decades [1].

To avoid the disadvantages of traditional breeding methods on egg production performance, such as time-consuming phenotyping, complicated procedures, and huge costs, many studies have been concentrated on molecular breeding that reveals candidate genes responsible for egg production traits, which helped lay a foundation for future improvements in egg production performance. Numerous genes related to egg reproduction traits have been identified by high-throughput sequencing technology. Previous research reported that *GARNL1* (GTP-activating Rap/Ran-GAP domain-like1) was identified as a candidate gene involved in regulating egg production in Ningdu Sanhuang chickens by suppressive subtractive hybridization [2]. Liao et al. identified an SNP site (ss1985401199) on chromosome Z, which was correlated significantly with egg production at the age of 25 to 45 weeks in Dongxiang blue-shelled and White Leghorn chickens [3]. A novel loci for egg production and quality traits in white leghorn and brown-egg dwarf layers was located on chromosome 7 [4]. However, egg-laying performance is a quantitative trait with lower estimated average heritability. The SNPs and QTLs identified by GWAS can’t deeply explain the genetic variation of egg production traits in a particular population or breed. The functional verification of candidate genes is still insufficient, and it is challenging to clarify the molecular mechanism of egg production differences.

Many studies have shown that RNA-seq has advantages in explaining complex traits such as egg-laying traits by identifying essential candidate genes. On this basis, studies have recently been focused on using RNA-Seq to study reproductive performance. Wang et al. obtained the transcriptome data of the hypothalamus and pituitary through RNA-seq to compare the egg-laying performance of high-yielding and low-yielding laying Chinese Dagu Chickens (CDC). Transcriptome analysis in the hypothalamus and pituitary identified 7 and 39 DEGs between high- and low-yielding CDC hens, respectively. In addition, functional annotation and pathway enrichment analysis showed glycosaminoglycan biosynthesis, phenylalanine metabolism, 2-oxocarboxylic acid metabolism, the glycosphingolipid biosynthesis-ganglion series, and local adhesion were significantly enriched in the hypothalamus and pituitary. Eight DEGs, including *PRDX6*, *TRIB2*, *OVCH2*, *CFD*, Peptidase M20, *SLC7A10*, and two other amino acid transporters, were identified as egg production biomarkers [5]. The study using comparative transcriptomic analysis between high and low egg-producing duck ovaries showed that a total of 843 DEGs was identified, *MC5R* and *APOD* were shown to influence egg production in Jinding ducks [6]. Transcriptome analysis of the hypothalamus and pituitary of turkey hens with low and high egg production revealed that the hypothalamic-pituitary-thyroid (HPT) axis and estradiol signaling played an essential role in regulating egg-laying performance [7]. Ma et al. compared the hypothalamus transcriptome profiles between Chinese indigenous chicken breeds with low egg-laying rates and commercial layers with relatively high egg-laying rates using RNA-seq, and a comprehensive analysis suggested that *RAC1*, *MRE11A*, *MAP7*, and *SOX5* were the potential candidate genes responsible for different egg-laying performance [8]. A recent study reported that the actin cytoskeleton pathway and three integrin family genes *ITGB2*, *ITGB5*, and *ITGA8* were identified in ovarian tissue, which provides signaling pathways and potential candidate genes for future genetic improvements of egg production in Longyan Shan-ma ducks [9]. However, the precise molecular mechanisms within the HPG axis regulating different egg-laying performances are still unclear. To further reveal the molecular mechanisms responsible for different egg-laying performances in ducks, transcriptomic analysis of the intact HPG axis-related tissues is required.

The formation process of poultry eggs is complicated and is mainly regulated by the HPG axis [10–12]. The vital endocrine glands include the hypothalamus, pituitary, and gonad, which secret various hormones and form the complex reproductive endocrine system that regulates reproductive activities. However, most previous studies have focused on the transcriptome changes of one or two tissues in the HPG axis, resulting in lacking systematic transcriptomic analyses about the HPG axis, which is impossible to investigate molecular mechanisms related to egg-laying differences thoroughly. Thus, it is necessary to analyze all HPG axis-related tissues’ transcriptomic profiles comprehensively.

According to the Food and Agriculture Organization of the United Nations (FAO), the number of ducks slaughtered is about 6.62 billion per year worldwide. The duck industry provides eggs and other agricultural products, so there is no doubt that duck breeding is an integral part of the farm industry. In the present study, the average egg-laying rate in HEP and LEP was different, and it was possible to improve the egg-laying rate of ducks by molecular breeding methods. Herein, we performed RNA-seq on all HPG axis-related tissues, including the hypothalamus, pituitary, ovary stroma, and F5 follicle membrane of individuals from HEP and LEP. This study aimed to identify candidate genes and molecular mechanisms responsible for the different egg-laying performances of ducks. These data will provide novel and comprehensive insights into the underlying molecular mechanisms within the HPG axis regulating different egg-laying performances in ducks.

## Results

### Production performance

**Fig. 1:**
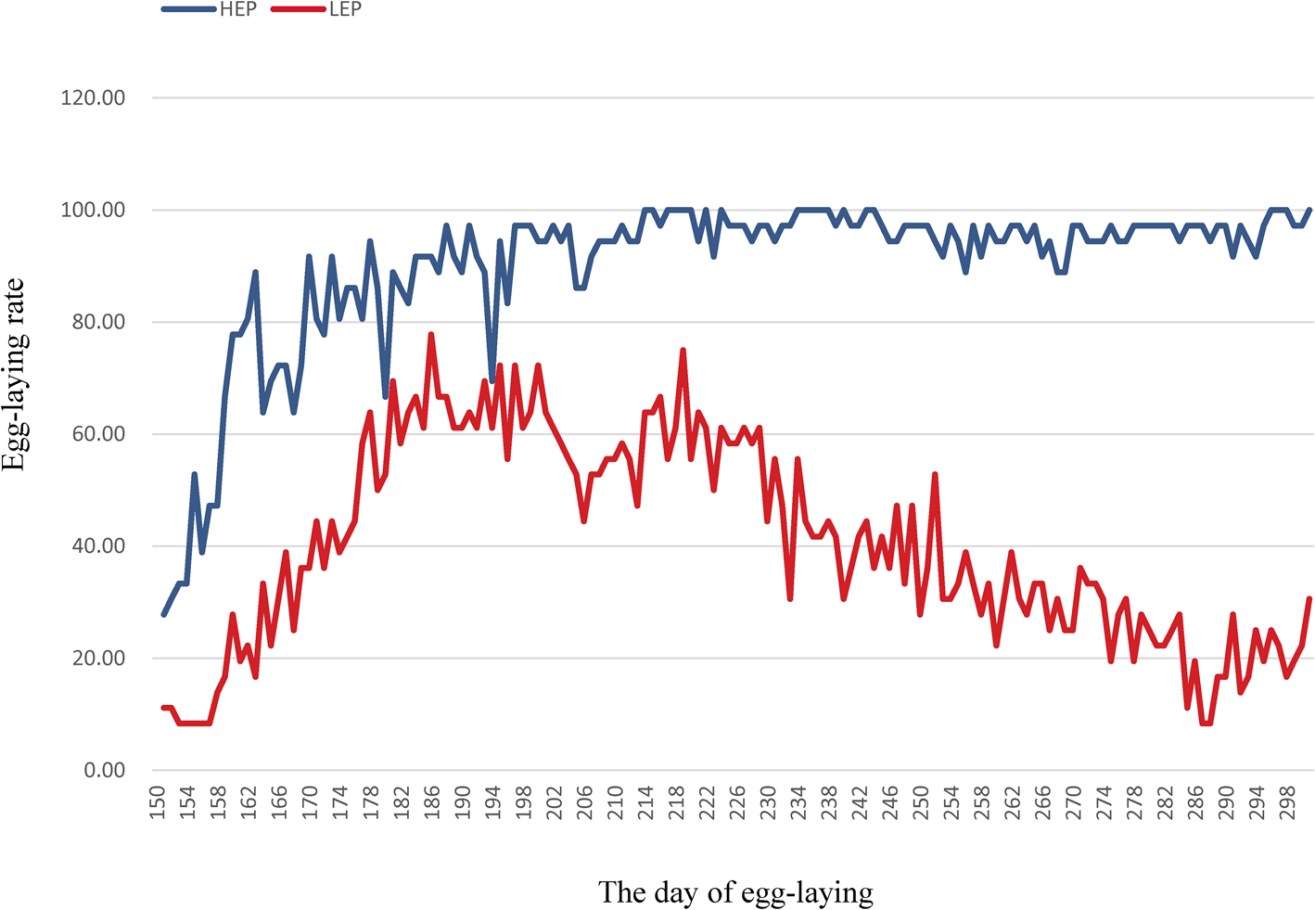
The curve of egg-laying rate in ducks. The abscissa represents the days of egg-laying. The ordinate represents the egg-laying rate. The blue curve represents the egg-laying rate of HEP. The red curve represents the egg-laying rate of LEP

Body weight at 300 days, age at first egg, egg number at 300 days and average egg production rate at 300 days ovarian weight, number of hierarchical follicles, and number of pre-hierarchical follicles (< 2 mm, 2 ~ 6 mm, 6 ~ 10 mm) of ducks were analyzed between HEP and LEP (Table 1). The results showed extremely significant differences (*P* < 0.01) in egg number at 300 days, average egg production rate at 300 days (Fig. 1 and Table 1) and significant differences (*P* < 0.05) in age at first egg and the number of hierarchical follicles between LEP and HEP groups. However, there were no significant differences in body weight at 300 days, ovarian weight, number of pre-hierarchical follicles in the two comparison groups (*P* > 0.05).

**Table 1:** Indicators related to egg production traits in HEP and LEP

Indicators	HEP	LEP	P
Body weight at 300 days (g)	2836.13±99.60	2880.86±202.43	0.588
Age at first egg (day)	152.00±5.81	161.71±9.46	0.031
Egg number at 300 days	140.36±3.28	56.89±20.25	0.001
Average egg production rate at 300 days (%)	87.15±22.34	35.33±19.30	0.001
Ovarian weight (g)	73.13±18.76	64.46±31.92	0.525
Number of hierarchical follicles	5.63±0.74	4.00±1.26	0.011
Number of pre-hierarchical follicles			
<2mm	0.84±0.29	0.91±0.25	0.605
2~6mm	2.89±1.28	3.70±0.91	0.199
2~6mm	3.76±1.86	4.57±2.23	0.456

### Identification of differentially expressed genes

**Fig. 2:**
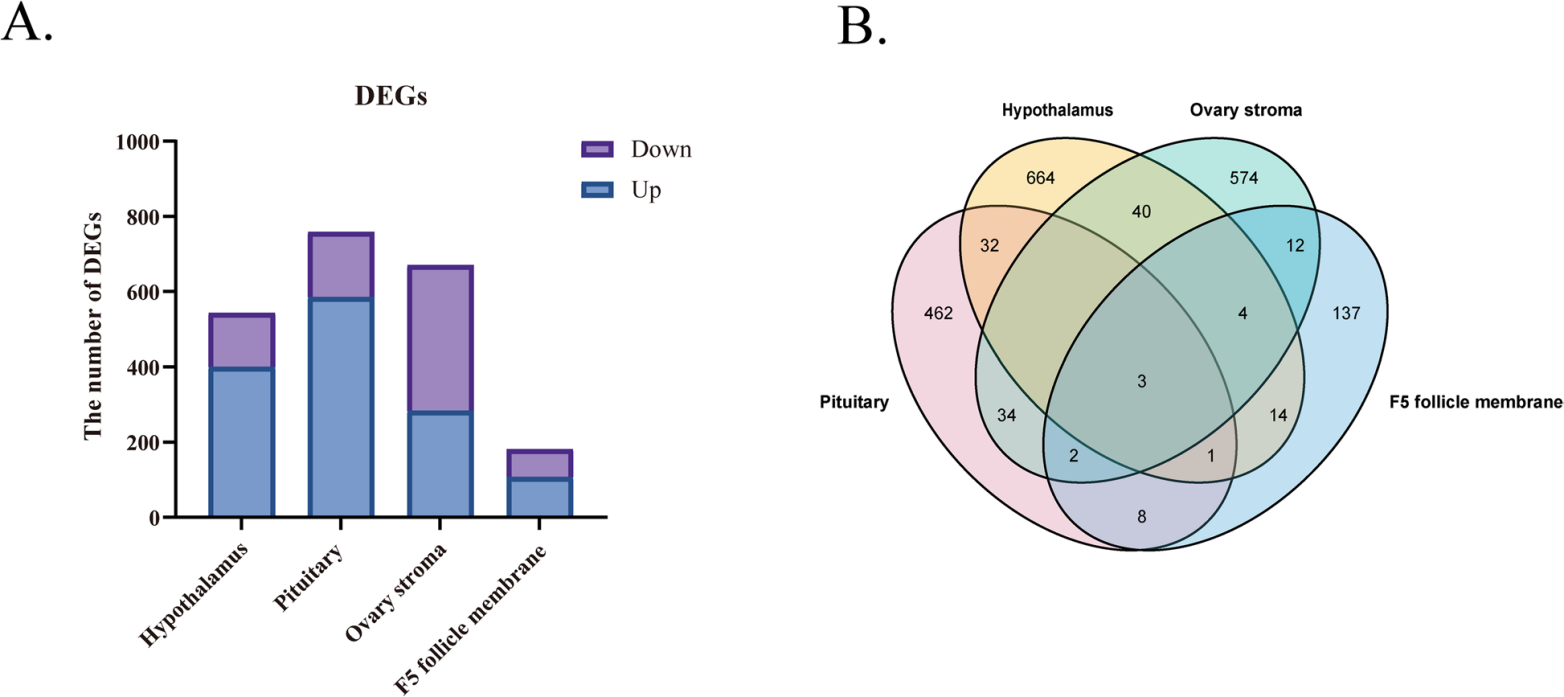
Overview of differentially expressed genes.** A** Histogram of the number of up-and downregulated genes in hypothalamus, pituitary, ovary stroma, and F5 follicle membrane between HEP and LEP. **B** Venn diagram of the DEGs from the HPG axis-related tissues between HEP and LEP

Principal component analysis (PCA) was conducted by GCTA software to analyze the hypothalamus, pituitary, ovary stroma, and F5 follicle membrane in HEP and LEP. Three biological replications at each sampling point are shown in (Figure S1). A total of 164.37 Gb clean bases were obtained from 24 samples through mRNA sequencing, with 92.71% of bases scoring Q30 and 88.26% ~ 90.43% of the sequencing reads were aligned to the duck reference genome (*Anas platyrhynchos*) (Table S1). DEGs were identified in the hypothalamus, pituitary, ovary stroma, and F5 follicle membrane between the HEP and LEP under the criteria of |Log_2_FC|≥ 1 and *P*-value < 0.05. A total of 2153 genes were differentially expressed in four HPG axis-related tissues, and among them, 1375 DEGs were up-regulated while 778 DEGs were down-regulated. Furthermore, analysis of the genes differentially expressed in four tissues indicated that the number of DEGs was much higher in the hypothalamus, pituitary, ovary stroma when compared to the F5 follicle. The number of DEGs in the hypothalamus, pituitary, ovary stroma and F5 follicle was 543 (400↑ up, 143↓ down), 759 (585↑ up, 174↓ down), 670 (283↑ up, 387↓ down), and 181 (107↑ up, 74↓ down), respectively (Table S2). Besides, in the hypothalamus, pituitary, and F5 follicle membrane, the number of up-regulated genes was higher than the down-regulated genes, while the number of up-regulated genes was lower than the down-regulated genes in ovary stroma (Fig. 2A). The Venn diagram (Fig. 2B) showed three common DEGs among two comparison groups, and the number of tissue-specific DEGs was 664, 462, 574, and 137 in the hypothalamus-pituitary, ovary stroma, and F5 follicle membrane, respectively.

### Enrichment analysis of Gene Ontology (GO) terms

**Fig. 3:**
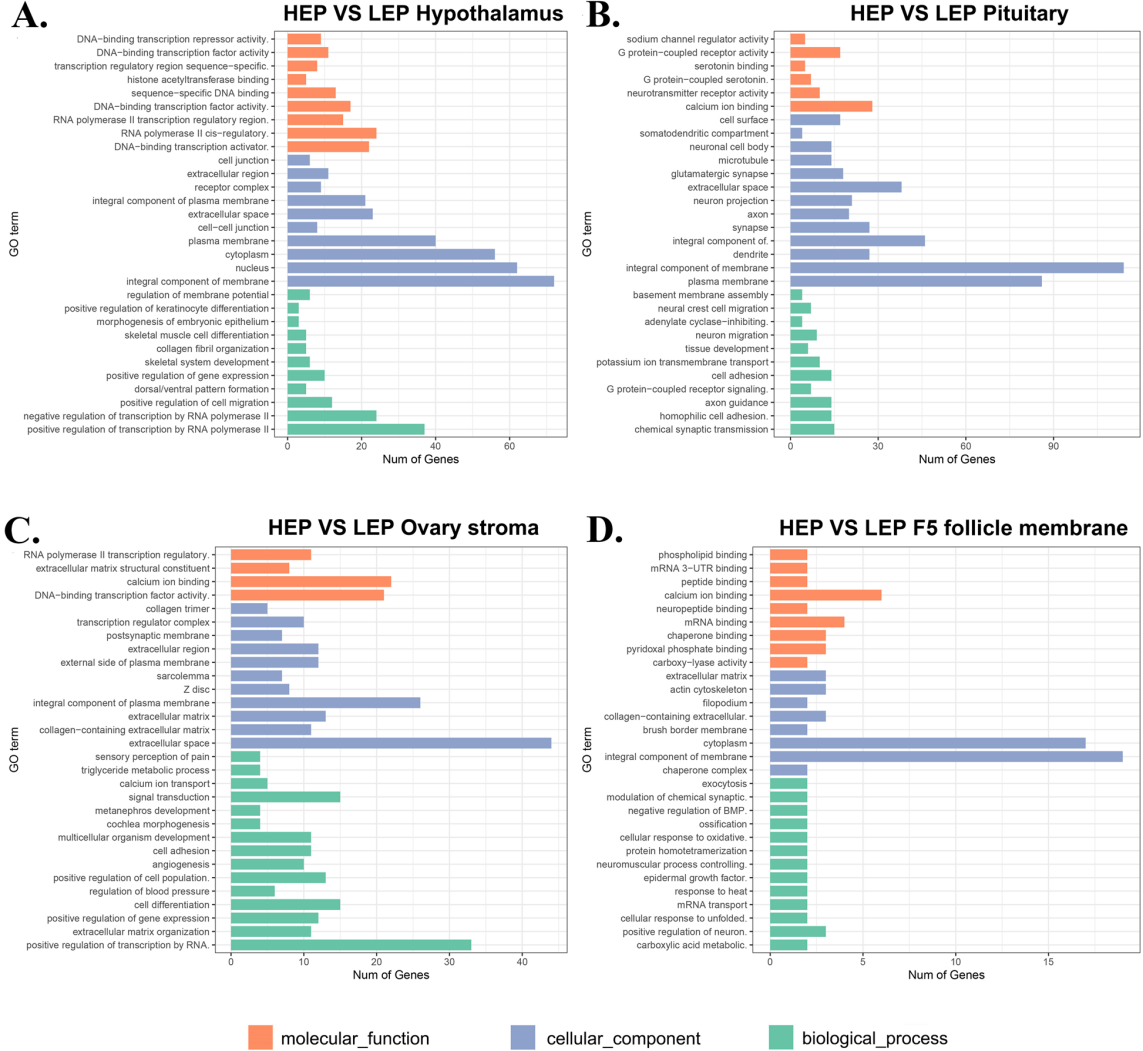
Enrichment analysis of Gene Ontology (GO) terms**.** GO classification of DEGs identified in the hypothalamus **A** pituitary **B** ovary stroma **C** and F5 follicle membrane **D** respectively. Orange indicates the molecular function, blue indicates cellular components, and green indicates biological processes

Enrichment analysis provides the most detailed GO terms and KEGG pathway information, which helps provide insights into the molecular mechanisms within the HPG axis underlying different egg production between HEP and LEP. A total of 2153 DEGs were enriched in 120 GO terms that were classified into three categories of biological process (BP), cellular component (CC), and molecular function (MF) (Fig. 3 and Tables S3). In four HPG axis related tissues, most DEGs were enriched in the "cellular component" category, the majority of DEGs in the hypothalamus identified between HEP and LEP were assigned to cellular components, such as integral components of membrane, nucleus, cytoplasm, plasma membrane, extracellular space, an integral component of the plasma membrane (Fig. 3A). In the pituitary, DEGs were mainly assigned to cellular components associated with an integral component of membrane, plasma membrane, extracellular space (Fig. 3B). In ovary stroma, the majority of DEGs were assigned to cellular components associated with extracellular space, an integral component of the plasma membrane, external side of the plasma membrane, extracellular region, and biological processes, such as positive regulation of transcription by RNA, cell differentiation, signal transduction, and molecular functions, such as DNA-binding transcription factor activity, calcium ion binding (Fig. 3C). In the F5 follicle membrane, DEGs were mainly assigned to cellular components associated with integral membrane and cytoplasm components and biological processes, such as calcium ion binding, mRNA binding (Fig. 3D).

### KEGG pathway enriched by the DEGs in HPG axis-related tissues

**Fig. 4:**
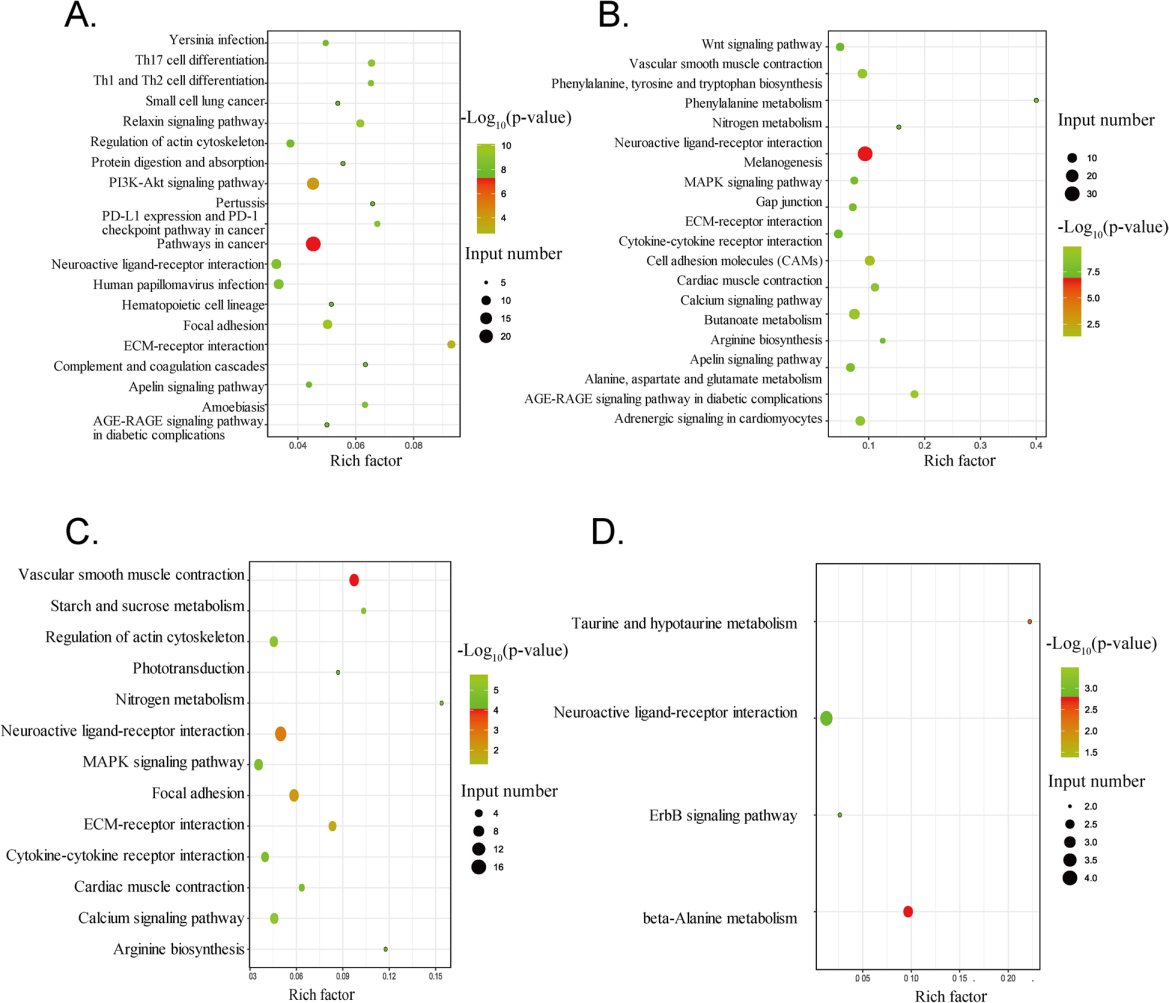
KEGG pathway enriched by the DEGs in HPG axis-related tissues. Top20 KEGG pathways enriched by the DEGs in the hypothalamus **A** pituitary **B** ovary stroma **C** and F5 follicle membrane **D** between HEP and LEP. The horizontal axis indicates the Rich factor, and the vertical axis shows the enrichment pathway. The circle size indicates the number of DEGs enriched in the pathway, and the point color corresponds to a different P-value range

The most significantly enriched pathways in HPG axis-related tissue were shown in (Fig. 4 and Table S4). There were 15 most significantly enriched pathways identified specifically in the hypothalamus. They were related to environmental information processing (PI3K-Akt signaling pathway), human diseases (Pathways in cancer, PD-L1 expression and PD-1 checkpoint pathway in cancer, Human papillomavirus infection, Amoebiasis, Pertussis, Yersinia infection, Small cell lung cancer, AGE-RAGE signaling pathway in diabetic complications), cellular processes (Relaxin signaling pathway), organismal systems (Th17 cell differentiation, Th1, and Th2 cell differentiation, Complement and coagulation cascades, Protein digestion and absorption, Hematopoietic cell lineage). In the pituitary, there were 7 tissue-specific enriched pathways involved in environmental information processing (Cell adhesion molecules, Wnt signaling pathway), metabolism (Alanine, aspartate and glutamate metabolism, Phenylalanine, tyrosine and tryptophan biosynthesis, Butanoate metabolism), organismal systems (Adrenergic signaling in cardiomyocytes), cellular processes (Gap junction). There were 4 most significantly enriched pathways identified specifically in the ovarian stroma, and they were related to metabolism (starch and sucrose metabolism, Arginine biosynthesis), environmental information processing (MAPK signaling pathway), organismal systems (Phototransduction). In the F5 follicle membrane, there were 3 tissue-specific enriched pathways involved in environmental information processing (beta-Alanine metabolism, ErbB signaling pathway) and metabolism (Taurine and hypotaurine metabolism). The neuroactive ligand-receptor interaction was the only pathway commonly enriched by DEGs identified in four HPG axis-related different tissues between HEP and LEP (Table 2).

**Table 2:** DEGs significantly enriched in neuroactive ligand-receptor interactions pathway

tissues	DEGs					
hypothalamus	KNG1↑	GPR83↓	PTGDR↓	PTGER4↑	PTRFR↓	LPAR3↑
	VIPR1↑	CCKAR↓	GABRA6↓	CHRNA5↑	CHRNA2↓	
pituitary	CHRM2↑	ADRA2B↑	HPH↑	HTR2A↑	HTR1F↑	HTR1D↑
	HTR1B↑	HTR1E↓	AGTR↑	GRP↑	EDN2↑	MC5R↑
	MTNR1B↑	S1PR1↑	INSL5↑	CALCB↑	CRH↑	CRHR2↑
	GIP↑	GLP1R↑	GRM5↑	GRM7↑	GABBR2↑	GABRA6↑
	GABRG2↑	CHRNA7↑	GRIK1↑	VIPR2↑		
ovary stroma	DRD1↑	DRD3↓	CHRM4↓	CCKBR↓	GALR1↓	GALR2↓
	POMC↑	TAC1↑	TAC4↑	AVPR1A↓	CGA↑	RXFP↓
	GABRA5↑	GABRG3↑	CHRNA4↓	GRIK3↑	P2RX1↓	
F5 follicle membrane	GABRG3↑	SSTR1↑	OPRN1↓			

### The gene expression profile clusters implied new regulatory pathways across HPG axis-related tissues

**Fig. 5:**
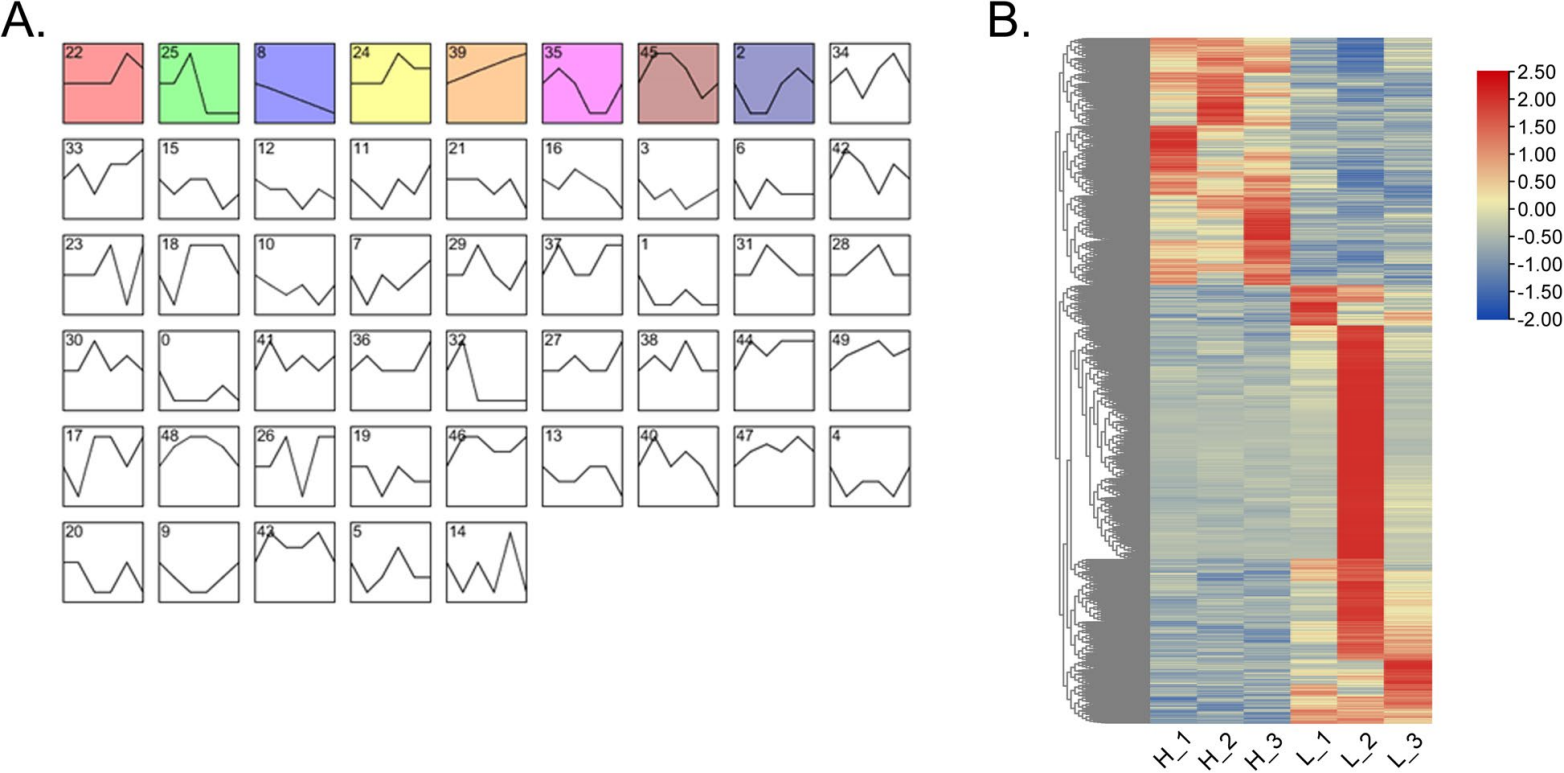
The gene expression profile clusters implied new regulatory pathways across HPG axis-related tissues. **A** The gene expression pattern of DEGs in the HPG axis-related tissues. Blocks represent genes with similar expression patterns. The vertical axis shows the gene expression level. Colored blocks indicate that the *P*-value < 0.05. The same color indicates that DEGs expression patterns in the HPG axis are similar. **B** Heatmap analysis of the expression profiles of the DEGs significantly enriched in clusters between HEP and LEP

Because genes with similar expression patterns may have a similar function or regulate common physiological processes. STEM (Short Time-series Expression Miner) software analyzed the gene expression profiles to identify the DEGs and potential pathways within the HPG axis that dominate egg production performance. All DEGs from HPG axis-related tissues were divided into 50 clusters (0–49), 8 of which reached significantly, including clusters 22, 25, 8, 24, 39, 35, 45, and 2 (Fig. 5A and Table S5). The expression profiles of clusters 8 and 39 were opposite among these clusters. The expression profiles of clusters 22, 24 and 25, 35, and 45 were similar. These DEGs were identified from each colorful cluster with similar expression patterns, which indicated they had a similar role in regulating egg-laying processes. As shown in Fig. 5B, these DEGs significantly enriched in all clusters displayed the same expression trend between HEP and LEP (Fig. 5B).

**Fig. 6:**
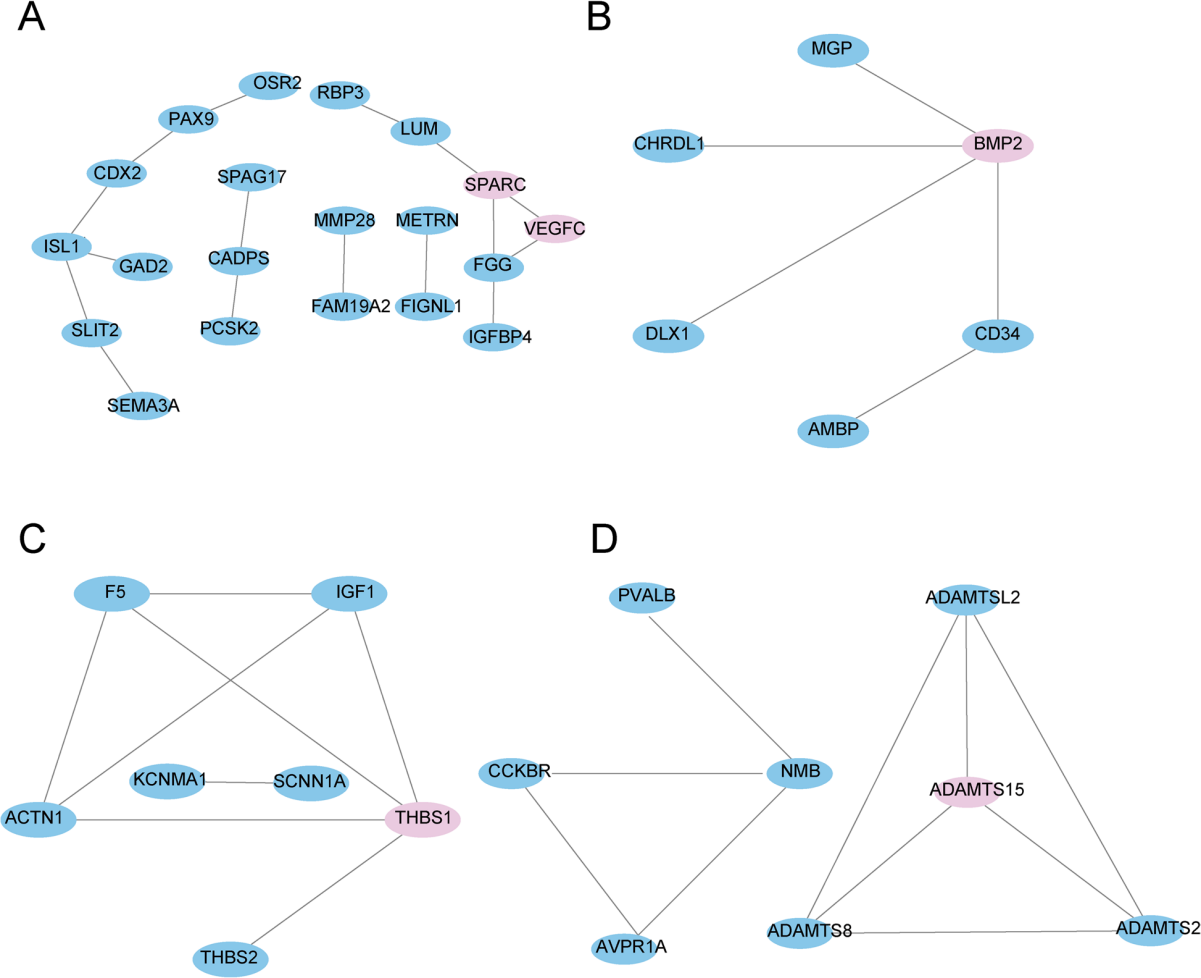
Map of protein–protein interaction networks in clusters 22(**A**), 25 (**B**), 35(**C**), and 8(**D**) between HEP and LEP*.* Nodes indicate proteins. The line shows the relationship between the two proteins, and the thickness of the line represents the close relationship between proteins

The DEGs encoding secretory proteins in the hypothalamus and pituitary and DEGs encoding target proteins in the ovary stroma and follicles were performed to identify the regulatory networks within the HPG axis-related tissues that mainly determine the egg production differences in these clusters. The present study focused on the relationship between secretory proteins in the hypothalamus and pituitary and targeted protein in the ovary stroma and follicle membrane. In general, clusters 22, 25, 35, 8, and 34 support the protein–protein interaction relationships (Fig. 6 and Table 3). In the hypothalamus, 7 DEGs (*SEMA3A*↑, *SPAG17*↑, *LUM*↓, *VEGFC*↑, *NMB*↓, *ADAMTS15*↓, and *WNT9A*↓) encoding secreted proteins were identified in clusters 22, 8, and 34, which has a relationship with 12 DEGs encoding target proteins in the ovary stroma and F5 follicle membrane. In the pituitary, 14 DEGs (*MMP28*↑, *SLIT2*↑, *RBP3*↑, *SPARC*↑, *BMP2*↓, *AMBP*↓, *ADAMTS2*↓, *THBS1*↓, *SHH*↑, *PCSK2*↑, *METRN*↑, *IGFBP4*↑, *SCNN1A*↓, and *WNT11*↑) encoding secretory proteins had a relationship with 15 DEGs encoding target proteins in the ovary stroma and F5 follicle membrane. In clusters 22, 25, 35, 8, and 34, *VEGFC*, *SPARC*, *BMP2*, *THBS1*, and *ADAMTS15* were considered key candidate DEGs within the HPG axis responsible for different egg production performance between HEP and LEP.

**Table 3:** The key DEGs enriched in HPG axis-related tissues

Tissue	Type	Cluster 22	Cluster 25	Cluster 35	Cluster 8
Hypothalamus	SP	SEMA3A↑,SPAG17↑			NMB↓
		LUM↑,VEGFC↑			ADAMTS15↓
Pituitary	SP&TP	MMP28↑,SLIT2↑	BMP2↓	THBS1↓	ADAMTS2↓
		RBP3↑	AMBP↓		
		SPARC↑			
	SP	PCSK2↑		SCNN1A↓	
		METRN↑			
		IGFBP4↑			
0vary stroma	TP	FAM19A2↑,ISL1↑	DLX1↓	KCNMA1↓	PVALB↓
		CDX2↑,PAX9↑	CHRDL1↓	F5↓	CCKBR↓
		OSR2↑,FGG↑	MGP↓	IGF1↓	AVPR1A↓
		CADPS↑,FIGNL1↑	CD34↓	ACTN1↓	ADAMTSL2↓
				THBS2↓	ADAMTS8↓
F5 follicle membrane	TP	GAD2↑			

## Discussion

The egg-laying rate is an important trait in the poultry industry and significantly differs among different individuals and breeds [6]. In the present study, the results showed extremely significant differences (*P* < 0.01) in egg number at 300 days, average egg production rate at 300 days and significant differences (*P* < 0.05) in age at first egg and the number of hierarchical follicles between LEP and HEP groups. In our study, the average egg production rate at 300 days of the HEP group was extremely significantly greater than that of the LEP group (87.15 ± 22.34% vs. 35.33 ± 19.30%, respectively). Thus, the animal models provided reliable data for the study of the egg-laying performance of ducks. Previous research reported a positive correlation between the number of follicles and the number of eggs produced in laying geese, which is mainly regulated by many important reproductive hormones [13]. We speculated that age at first egg and the number of hierarchical follicles might be related to egg production.

The phenotypic indicators of egg production traits are mainly regulated by many important reproductive hormones secreted by the HPG axis [14]. Previous studies have focused on the hypothalamus, pituitary, or ovaries to reveal the molecular mechanism associated with egg production performance in ducks [15–17]. In the present study, the whole transcriptome of the HPG axis was constructed to reveal the regulatory mechanism of DEGs involved in high and low egg production ducks. A total of 543, 759, 670, and 181 DEGs were identified in the hypothalamus, pituitary, ovarian stroma, and F5 follicle wall between HEP and LEP, respectively. To the best of our knowledge, this is the first report to provide the complete transcriptomic profile of the HPG axis regulating the egg production performance by RNA-seq in duck.

GO annotation and KEGG analyses were performed to investigate the DEGs’ biological functions. It was worth noticing that neuroactive ligand-receptor interaction was the only common pathway identified in the hypothalamus, pituitary, ovary stroma, and F5 follicle membrane. Transcriptome studies in pigs [18], goats [19], zebrafish [20], and poultry [6, 21] have also demonstrated that the neuroactive ligand-receptor interaction pathway is involved in regulating reproductive activities. We found that the expression of DEGs was significantly different in the neuroactive ligand-receptor interaction pathway between HEP and LEP. The neuroactive ligand-receptor interaction pathway has also been reported to regulate the egg production performance of chickens [21] and ducks [6]. Thus, the neuroactive ligand-receptor interaction pathway may be considered as an underlying molecular pathway within the HPG axis regulating different egg-laying performance between HEP and LEP.

Gene expression profiles analysis of all DEGs was performed to gain further insight into the key molecules and the underlying regulatory mechanisms within the HPG axis responsible for different egg production. We obtained 5 clusters in which DEGs were statistically significant and have similar gene expression patterns. These data suggested that ovaries and the follicle membrane may respond to the regulation of secreted proteins from the hypothalamus and pituitary. We speculated that underlying regulation across the HPG axis might be found in the present study. Thus, PPI network analysis of the DEGs encoding secretory proteins and target proteins was carried out to comprehensively investigate the potential regulatory pathways in the HPG axis. *VEGFC*, *SPARC*, *BMP2*, *THBS1*, and *ADAMTS15* are key candidate DEGs because they were located in a core position in the regulatory networks.

We found that several members from the *ADAMS* genes family constructed a regulatory network within HPG axis-related tissues in cluster 8, *ADAMTS15* is located at the core of the PPI network in cluster 8. Previous research reported that The *ADAMTS* gene family is essential for reproductive processes such as ovulation [22, 23]. The gene expression levels of several *ADAMTS* proteases influenced regulating the surge of LH/FSH in the receptor, indicating that the protease family can remodel ovulatory follicles in preparation for ovulation [24]. In our study, *ADAMTS15* and *ADAMTS2* secreted by the hypothalamus and pituitary, they interact with the target proteins *ADAMTSL2* and *ADAMTS8* identified in the ovarian stroma. Therefore, we speculated that *ADAMTS15* might play a role in egg-laying performance by regulating the development of ovarian follicles.

In cluster 25, *BMP2* located the core position in the regulatory network within HPG axis-related tissues. Previous research reported that *BMP2* can enhance the synthesis of estradiol-induced by *FSH* in rat ovarian granulosa cells [25]. In addition, *BMP2* can also promote estradiol synthesis in ovarian granulosa cells and inhibit progesterone production [26]. In this study, *BMP2* was identified as a secretory protein in the pituitary, which had a relationship with target proteins. Gene expression of these genes was down-regulated between two comparison groups. It indicated that *BMP2* might be involved in the egg-laying performance by regulating development of follicles and ovulation.

In pituitary and ovary stroma, DEGs were closely related to angiogenesis in clusters 22 and 35. Sufficient blood vessel formation involves important physiological processes in the ovaries, such as the corpus luteum, follicle formation, and ovulation. *VEGFC* (vascular endothelial growth factor C) [27, 28] and *SPARC* (secreted protein acidic and rich in cysteine gene) [29] are mainly involved in angiogenesis. Previous research reported that *VEGFC* is one of the most important regulators of angiogenesis, which plays an important regulatory role in the development and maintenance of the blood vessels around the follicle and the blood vessels in the corpus luteum [30]. *SPARC* is a widely distributed secreted matrix protein that interacts with cytokines *VEGFC*, etc., and affects cell proliferation by regulating the activity of cytokines[31]. *SPARC* and *VEGFC* form a dynamic network regulating angiogenesis and vascular degeneration, eventually affecting normal follicular development [31, 32]. In this study, *VEGFC* and *SPARC* were identified as secreted proteins in cluster 22, and their expression profiles were similar. In addition, *VEGFC* and *SPARC* are located in the center of the PPI network (the network contains 11 DEGs encoding secretory proteins in the hypothalamus and pituitary, and 9 DEGs encoding target proteins in the ovary stroma and follicles). Therefore, we speculated that *VEGFC* and *SPARC* in the hypothalamus and pituitary constructed a dynamic regulation network regulating egg production performance. In addition, DEGs related to angiogenesis were identified in cluster 35. *THBS1* can inhibit follicular angiogenesis and directly induce apoptosis of mouse granulosa cells [33]. Female mice with *THBS1* gene deletion have reduced litter size and have reproductive dysfunction [34]. *THBS1* was located in the center of the PPI network, we speculated that *THBS1* might play an important role in egg-laying performance by regulating the development of angiogenesis.

At the neuroendocrine level, egg production is regulated by the HPG axis [35]. Reproductive hormones secreted by the hypothalamus, pituitary, and gonads maintain poultry’s relatively stable reproductive endocrine system. Neuroendocrine cells located in the hypothalamus can synthesize GnRH, enter the pituitary, and then bind specifically to the GnRHR. The physiological processes can stimulate the synthesis and secretion of LH and FSH to regulate the secretion of gonadal steroids and the occurrence of gametes [36–38]. In the present study, the gene expression of classic reproductive hormones including GnRH, LH, and FSH did not change, suggesting that the GnRH-FSH/LH pathway may not be the only regulation mechanism determining egg-laying performance in ducks. Our findings will complement the classical regulatory pathways in the HPG axis.

## Conclusion

In conclusion, this is the first report to provide the complete transcriptomic profile of the HPG axis regulating the egg production performance by RNA-seq in ducks. Comprehensive analysis suggested that the neuroactive ligand-receptor interaction pathway, particularly 5 candidate DEGs including *VEGFC*, *SPARC*, *BMP2*, *ADAMTS15*, and *THBS1*, were crucial for egg production in ducks. Findings in our research could provide novel insights into the underlying molecular mechanisms regulated by the HPG axis in HEP and LEP ducks. The potential genes identified in this study can be used as a selection marker in ducks to increase laying performance.

## Materials and methods

### Experimental animals

360 female ducks (Tianfu Nonghua Ma duck GF2 line) were obtained from the Waterfowl Breeding Experimental Farm of Sichuan Agricultural University, Ya’an, Sichuan province, China. These ducks were hatched in the same batch. The ducklings were fed and managed under the same environmental conditions with free access to feed and water throughout the brooding, maturation, and laying period. The immunization procedure followed the waterfowl immunization procedure developed by the local animal husbandry and veterinary department. Experimental ducks were raised on the ground, and then they were transferred to a single cage at 120 days of age. The egg numbers of individuals from 130 to 300 days were recorded daily. At the age of 300 days, the group’s average egg production number and standard deviation were calculated according to the egg production records. In the present study, the ducks from the highest 10% egg production were defined as the high egg production group (HEP), and the ducks from the lowest 10% production were defined as the low egg production group (LEP).

### Sample collection

**Fig. 7:**
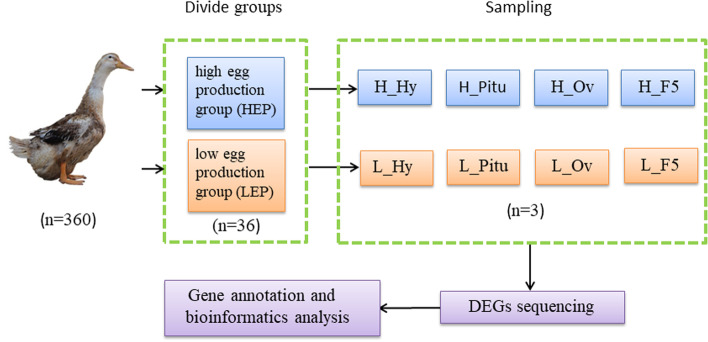
The entire experimental design and sequencing process. H_Hy, H_Pitu, H_Ov, and H_F5 represent hypothalamus, pituitary, ovary stroma, and F5 follicle membrane of ducks in the high egg production group (HEP). L_Hy, L_Pitu, L_Ov, and L_ F5 represent the corresponding tissues of ducks in the low egg production group (LEP)

Six healthy ducks were selected from the HEP and LEP group according to the same laying patterns as the final experimental animals. The reproductive traits such as body weight at 300 days, age at first egg, egg number and average egg production rate at 300 days, ovarian weight, number of hierarchical follicles, and number of pre-hierarchical follicles, were recorded for each duck (Table 1). All selected ducks were euthanized by inhaling carbon dioxide and cervical dislocation after fasting for about 12 h. The hypothalamus, pituitary, ovary stroma, and F5 follicle membrane were collected, respectively, and then they were rapidly frozen in liquid nitrogen. All samples were stored at -80 °C for RNA-Seq. The entire experimental design and sequencing process was shown in (Fig.7).

### RNA preparation

Total RNA was extracted from each hypothalamus, pituitary, ovary stroma, and F5 follicle membrane using Trizol reagent (Invitrogen, CA, USA) following the manufacturer’s instructions. The RNA quality and concentration were measured using the Qubit1 RNA Assay Kit with the Qubit1 2.0 Fluorometer (Life Technologies, CA, USA) and the RNA Nano 6000 Assay Kit with the Agilent Bioanalyzer 2100 system (Agilent Technologies, CA, USA). RNA that meets the criteria (A260/A280 ≥ 1.8, A260/A230 ≥ 2.0, and RNA integrity number > 7.0) was selected for subsequent analysis.

### Library construction and sequencing

The cDNA sample from HEP and LEP ducks was prepared for sequencing as a cDNA library. Poly (A) + mRNA was purified with mRNA capture beads, and the mRNA was randomly divided into small fragments by divalent cations in the fragmentation buffer. RNA short fragments were used as templates to synthesize the first-strand cDNA using random hexamer primers. Second strand cDNA was synthesized using RNase H and DNA polymerase I. Short cDNA fragments were purified with AMPure XP beads. The library fragments were purified and eluted with elution buffer, then the terminal repair, add poly (A), and the adapter was implemented. Then PCR amplification was performed, 300 ~ 500 bp fragments were selected to create a cDNA library.

The RNA libraries were prepared using the VAHTS mRNA-seq V3 Library Prep Kit for Illumina® according to the product instructions [39, 40]. The quality and insert size of the cDNA libraries were checked using the Agilent 2100 Tape Station system (Agilent). Then, libraries were sequenced on the Illumina sequencing platform (Nova Seq 6000 Illumina), and 150 bp paired-end reads were generated. The original image file was obtained, and then the base recognition and conversion were carried out to obtain the Raw sequencing data. Filter the original sequencing data according to certain standards to obtain high-quality clean data after quality control. Fast QC analyzed raw reads for quality, and high-quality reads with Q > 20 were obtained using NGS Toolkits (version: 2.3.3) [41]. The duck genome file (IASCAAS_Peking Duck_PBH1.5, GCF_003850225.1) and genome annotation file (PBH1.5. Gff3) were downloaded from the NCBI database (https://www.ncbi.nlm.nih.gov/) to build a genome in Hisat 2 file index. Specifically, all paired clean transcriptome reads were mapped to the duck genome (IASCAAS_Peking Duck_PBH1.5, GCF_003850225.1) using the HISAT2 (V2.1.0) software. Samtools software converts the Sam files obtained by mapping into bam files and sorts them. String Tie was used to assemble transcripts, quantitatively express genes, and estimate gene expression abundance. Gffcompare detects gene annotation and transcript assembly. DEGs between the two groups were identified using the DESeq2 R package [42]. Gene expression was calculated by FPKM method and TPM method. |Log_2_FC|≥ 1 and *P*-value < 0.05 were set as the threshold for significantly differential expression.

### Function annotation of DEGs

Gene ontology enrichment analysis software tools (GOEAST) [43] were used to analyze the Gene Ontology (GO) functions. Biological process, molecular function, and cellular component were used to annotate the main function of DEGs in GO enrichment analysis. KOBAS3.0 [44]was used to analyze the Kyoto Encyclopedia of Genes and Genomes (KEGG) functions [45]. It is generally considered that GO terms and KEGG pathways with corrected *p*-values ≤ 0.05 are significantly.

### Gene expression pattern analysis

Gene expression patterns of HEP and LEP were clustered by STEM software[46]. The ’Log normalize data’ method was used to convert expression quantity, and the other options were set to default values because they have been proven to provide the optimal results with biological and simulated data [47]. The expression level was the previously calculated FPKM value, the *P*-value < 0.05 in the clustered profile, which was considered significant [48].

### Protein interaction network analysis

The protein–protein interaction (PPI) network of DEGs encoding secreted proteins in the hypothalamus and pituitary, as well as those encoding target proteins in the ovary stroma and F5 follicle membrane, were built up using the STRING protein interaction database (http://string-db.org/) by calculating the combined score (threshold: score > 0.4). The node represents a protein, and the edge represents an interaction between two proteins in the PPI network. Cytoscape software version 3.5.1 (http://www.cytoscape.org/) was used to realize data visualization.

### Statistical analysis

Data were analyzed using the SPSS statistical software version 20.0 (IBM Corp., Armonk, New York). In this study, the means of the phenotypic indicators between HEP and LEP groups were subjected to ANOVA testing, including body weight at 300 days, age at first egg, egg number at 300 days, average egg production rate at 300 days, ovarian weight, number of hierarchical follicles, number of pre-hierarchical follicles. The means were assessed for significance by Tukey’s test, and t-tests were used to analyze the relevance between the two groups. The data were presented as Mean ± SEM, and *P* < 0.05 was used as a significant statistical difference.

## Supplementary Information

**Additional file 1.** 

**Additional file 2:** **Table S2. **DEGs identified in hypothalamus, pituitary, ovary stroma, and F5 follicle membrane between HEP and LEP.

**Additional file 3:** **Table S3. **GO enriched in hypothalamus, pituitary, ovary stroma, and F5 follicle membrane between HEP and LEP.

**Additional file 4:** **Table S4. **KEGG enriched in hypothalamus, pituitary, ovary stroma, and F5 follicle membrane between HEP and LEP.

**Additional file 5:** **Table S5. **Gene expression pattern analysis.

## Data Availability

The RNA sequencing raw data was previously released in the GSA database (https://bigd.big.ac.cn/gsa/browse/CRA005053; Accession: CRA005053).

## Abbreviations

*ADAMTS15*: A disintegrin and metalloproteinase with thrombospondin motif-15
BP: Biological process
*BMP2*: Bone morphogenetic protein-2
CC: Cellular component
DEGs: Differentially expressed genes
GO: Gene Ontology
HEP: High egg production
HPG: Hypothalamic-pituitary–gonadal
*IGF-1*: Insulin-like growth factor-1
KEGG: Kyoto Encyclopedia of Genes and Genomes
LEP: Low egg production
MF: Molecular function
PPI: Protein–Protein Interaction
*SPARC*: Secreted protein acidic and rich in cysteine
*THBS1*: Thrombospondin-1
*VEGFC*: Vascular endothelial growth factor C
