# Supplementary material for: Transcriptomic analyses of the HPG axis-related tissues reveals potential candidate genes and regulatory pathways associated with egg production in ducks
Source: BMC Genomics. 2022 Apr 8;23:281. doi: 10.1186/s12864-022-08483-y (PMC8991983; doi:10.1186/s12864-022-08483-y)
Supplement: Supplementary file 1 — Additional file 1. [file 12864_2022_8483_MOESM1_ESM.docx]

**Supplementary files**

**Transcriptomic analyses of the HPG axis-related tissues reveals potential candidate genes and regulatory pathways associated with egg production in ducks**

Xiping Yan^^[[1]](#footnote-1)^^, Hehe Liu^2^*, Jiwei Hu^2^, Xingfa Han^1^, Jingjing Qi^2^, Qingyuan Ouyang^2^, Bo Hu^2^, Hua He^2^, Liang Li^2^, Jiwen Wang^2^, and Xianyin Zeng^1^*

A Department of Engineering and Applied Biology, College of Life Science, Sichuan Agricultural University, Ya'an, Sichuan, 625014, P.R. China.

^2^ Farm Animal Genetic Resources Exploration and Innovation Key Laboratory of Sichuan Province, Sichuan Agricultural University, Chengdu, Sichuan, 611130, P.R. China.

Short title: RNA-seq of HPG axis-related tissues in ducks

Xiping Yan and Hehe Liu contributed equally as the first authors.

*To whom correspondence should be addressed.

Xianyin Zeng, xyzeng@sicau.edu.cn

Hehe Liu, [liuee1985@sicau.edu.cn](mailto:liuee1985@sicau.edu.cn)

**Supplementary files**

**Figure S1. Principal Component Analysis.** PCA was conducted on the transcriptome information of each sample using the GCTA tool. PCA in the hypothalamus **A**, pituitary **B**, ovary stroma **C**, and F5 follicle membrane **D**, respectively. The orange dots represent individuals from HEP, and the blue dots represent individuals from LEP.


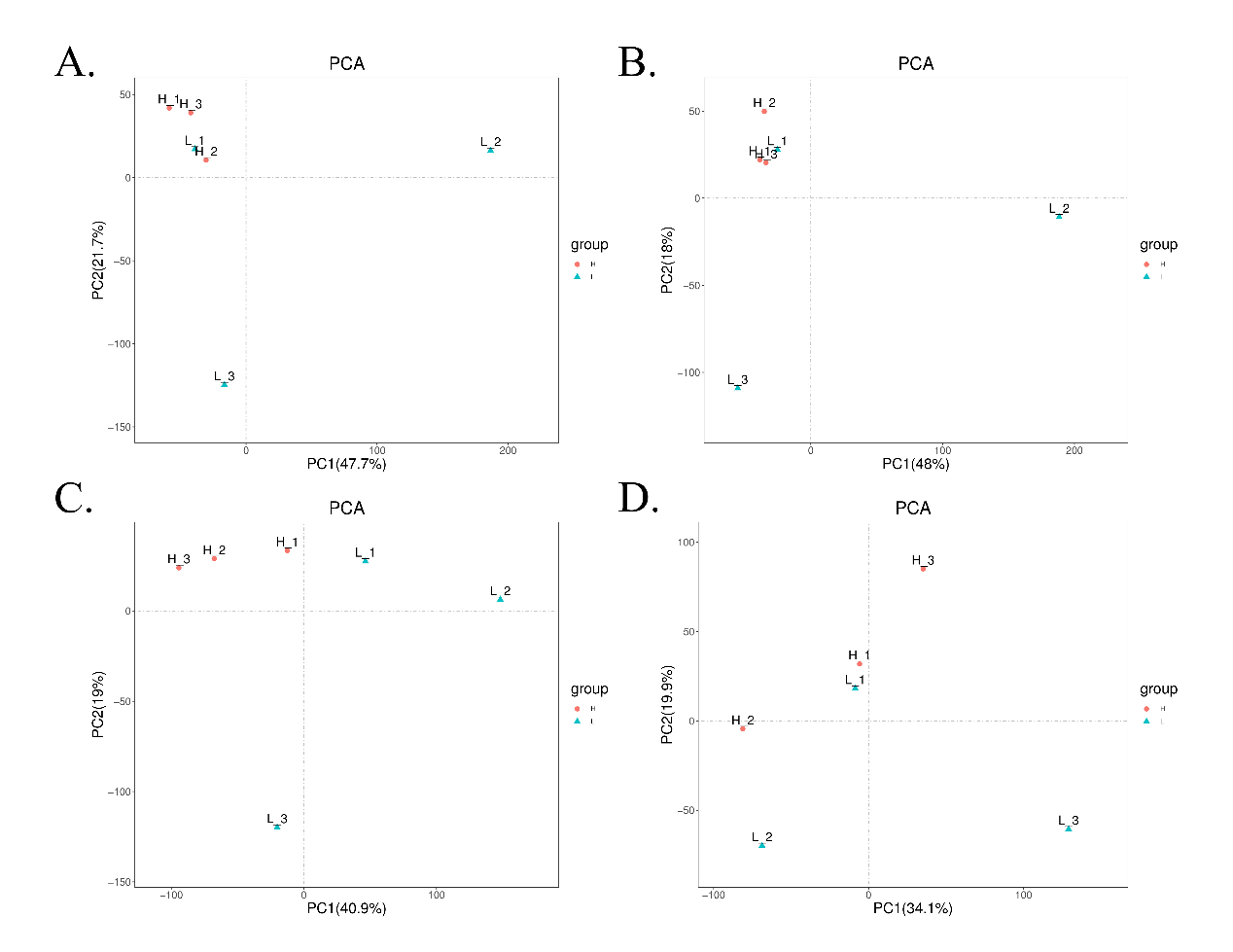


**Table S1.** Basic information of mRNA sequencing data of all samples in this study.

| Sample ID | Clean Reads | Base (bp) | Q20(%) | Q30(%) | GC (%) | Mapping rate (%) |
| --- | --- | --- | --- | --- | --- | --- |
| H_Hy | 24,348,220 | 7,278,493,799 | 97.06 | 92.46 | 48.86 | 90.43 |
| L_Hy | 21,386,686 | 6,389,476,354 | 97.27 | 92.89 | 47.67 | 89.86 |
| H_Pitu | 23,618,912 | 7,061,651,571 | 97.03 | 92.33 | 48.29 | 89.73 |
| L_Pitu | 23,544,179 | 7,031,985,584 | 97.09 | 92.52 | 48.24 | 89.33 |
| H_Ov | 22,763,444 | 6,804,850,152 | 97.17 | 92.63 | 49.67 | 89.70 |
| L_Ov | 23,081,170 | 6,897,643,587 | 97.40 | 93.00 | 49.43 | 88.26 |
| H_F5 | 23,129,598 | 6,911,493,108 | 97.19 | 92.73 | 49.11 | 90.09 |
| L_F5 | 22,991,404 | 6,871,328,949 | 97.39 | 93.09 | 49.40 | 89.69 |

**Table S2.** DEGs identified in hypothalamus, pituitary, ovary stroma, and F5 follicle membrane between HEP and LEP.

**Table S3.** GO enriched in hypothalamus, pituitary, ovary stroma, and F5 follicle membrane between HEP and LEP.

**Table S4.** KEGG enriched in hypothalamus, pituitary, ovary stroma, and F5 follicle membrane between HEP and LEP.

**Table S5.** Gene expression pattern analysis.

1. [↑](#footnote-ref-1)
